# GNRHR-related central hypogonadism with spontaneous recovery – case report

**DOI:** 10.1186/s13052-022-01377-5

**Published:** 2022-11-12

**Authors:** Darja Šmigoc Schweiger, Maja Davidović Povše, Katarina Trebušak Podkrajšek, Tadej Battelino, Magdalena Avbelj Stefanija

**Affiliations:** 1 Department of Pediatric Endocrinology, Diabetes and Metabolism, University Medical Centre Ljubljana, University Children’s Hospital, Ljubljana, Slovenia; 2 Community Health Centre Novo Mesto, Novo Mesto, Slovenia; 3 Clinical Institute of Special Laboratory Diagnostics, University Medical Centre Ljubljana, University Children’s Hospital, Ljubljana, Slovenia; 4 Faculty of Medicine, University of Ljubljana, Ljubljana, Slovenia

**Keywords:** Congenital hypogonadotropic hypogonadism, Gonadotropin-releasing hormone receptor, Testosterone, Reversial, Case report

## Abstract

**Background:**

Congenital hypogonadotropic hypogonadism (CHH) is a clinically and genetically heterogeneous disease characterized by absent or incomplete puberty and infertility. Clinical characteristics are secondary to insufficient gonadotropin secretion, caused by deficient gonadotropin-releasing hormone (GnRH) production, secretion, or action. Loss-of-function variants of the gonadotropin-releasing hormone receptor (GNRHR) are associated with CHH without anosmia. CHH was previously considered a permanent condition, but in the past two decades, cases of spontaneous recovery of CHH have been reported. The reversal of hypogonadism in CHH is currently unpredictable, and can happen unnoticed.

**Case presentation:**

The male proband was diagnosed with CHH due to compound heterozygosity for two previously reported pathogenic missense variants in the *GNRHR* gene, NM_000406.2:c.416G > A (NP_000397.1:p.Arg139His) and c.785G > A (p.Arg262Gln) at 16 years of age. In addition to arrested partial puberty, he had a low testosterone level, gonadotropins in the range of early puberty, and a normal inhibin B level. A therapy with increasing doses of intramuscular testosterone undecanoate was received for 2.5 years, while there was no change in testicular volume. At the age of 19 years, testosterone supplementation was interrupted. During the next two years, he had spontaneous pubertal development to achieve a testicular volume of 20 mL, with normal adult levels of gonadotropins and testosterone.

**Conclusions:**

Genetic diagnostics can help discriminate congenital hypogonadotropic hypogonadism, deserving therapeutic intervention, from the self-limited constitutional delay of growth and puberty (CDGP). Patients with *GNRHR* associated hypogonadism can experience spontaneous recovery of the hypothalamic-pituitary–gonadal axis. Spontaneous testis enlargement in patients with central hypogonadism not taking gonadotropins or pulsatile GnRH therapy can indicate recovery of hypogonadism.

## Background

The most common cause of delayed puberty is a constitutional delay of growth and puberty (CDGP) [1], but a small proportion of patients has congenital hypogonadotropic hypogonadism (CHH). CHH is a clinically and genetically heterogeneous disease characterized by absent or incomplete puberty and infertility [2]. Clinical characteristics are secondary to insufficient gonadotropin secretion, caused by deficient gonadotropin-releasing hormone (GnRH) production, secretion, or action. Incidence is 1–10 cases per 100 000 births [3, 4] and it is two times more prevalent in males than females [2]. CHH can be isolated with otherwise normal pituitary function or associated with other developmental disorders (cleft lip or palate, dental agenesis, sensorineural deafness, renal agenesis, bimanual synkinesis, skeletal anomalies) or other pituitary deficiencies. CHH is in about half of cases associated with anosmia or hyposmia when it is classified as Kallmann's syndrome. CHH without anosmia is classified as normosmic CHH (nCHH). Sporadic and familial cases of CHH were described. CHH can be inherited in either X – linked or autosomal modes [5]. Molecular-genetic aetiology is identified in about 50% of CHH patients, demonstrating high genetic heterogeneity with more than 30 different genes being involved [2, 6, 7]. The majority of genes associated with CHH regulate GnRH neuron development. A small subset of genes regulates GnRH production, secretion, or action [5]. GnRH and its receptor (GnRHR) are central regulators of puberty. Loss-of-function variants of the *GNRHR* are associated with CHH without anosmia [8, 9]. GnRHR is a G protein-coupled receptor expressed on gonadotropic cells of the pituitary gland. Its activation regulates the synthesis and secretion of luteinizing hormone (LH) and follicle-stimulating hormone (FSH) [8, 10]. In a large cohort of 863 CHH patients, *GNRHR* variants had an overall prevalence of 4% [11]. CHH due to *GNRHR* variants follows autosomal recessive inheritance. In some patients only monoallelic *GNRHR* pathogenic variants are identified either alone or in combinations with variants in other genes, demonstrating complex and oligogenic inheritance [11]. CHH was previously considered to be a permanent condition, but in the past two decades, cases of spontaneous recovery of CHH have been reported. The latest cohort study estimated lifetime incidence of reversal up to 22% [12].

## Case presentation

Clinical and biochemical characteristics were obtained from the patient's medical records. Anthropometric Z-scores were calculated using the LMS method and the British 1990 reference. Serum levels of LH, FSH, and testosterone were measured using the Immulite 2000 immunoassay system (Siemens, Germany), and inhibin B was measured using the Gen II ELISA assay (Beckman Coulter Inc., USA). GnRH stimulation test was performed using gonadorelin 100 mcg intravenously, serum LH and FSH were measured at 0', 20', 30' and 60' min. Bone age was evaluated based on Greulich and Pyle Atlas (GP) bone age determination system, 2nd edition. The clinical data are summarized in Table 1.

**Table 1:** Patient’s characteristics and clinical course

	**Baseline**	**After 1 year of testosterone th**	**After 2 years of testosterone th**	**2 years after cessation of testosterone th**
**Age (years)**	16	17	18	20
**Bone age (years)**	14.7	/	/	/
**Bone age SDS**	-1.48	/	/	/
**Height (cm)**	178.8	182	185	188.8
**Height SDS**	0.62	0.72	1.11	1.64
**Weight (kg)**	88.2	84.65	90.4	99
**Weight SDS**	2.11	1.63	1.92	2.39
**BMI (kg/m**^**2**^**)**	27.59	25.56	26.4	27.79
**BMI SDS**	2.13	1.48	1.55	1.73
**Testicular volume (mL)**	4	4	4	20
**Tanner stage**	2–3	3	4	4–5
**Basal LH (IU/L)** LoD 1.8 IU/L ≤ 7% CV	0.9	/	/	3.8
**Basal FSH (IU/L)** LoD 0.6 IU/L ≤ 7% CV	2.2	/	/	10.4
**Peak stimulated LH (IU/L)**	6.58	/	/	18.2
**Peak stimulated FSH (IU/L)**	3.58	/	/	15.6
**Testosterone (nmol/L)** LoD 0.17 nmol/L ≤ 10% CV	0.8	11.8	18.3	23.6
**Inhibin B (ng/L)** LoD 2.91 ng/L ≤ 6.6% CV	85.8	/	126.6	99.3

*SDS* Standard deviation score, *BMI* Body mass index, *LH* Luteinizing hormone, *LoD* Limit of Detection, *CV* Inter-assay coefficient of variation, *FSH* Follicle-stimulating hormone

The male proband presented at 16 years of age with clinical signs of hypogonadism, i.e. microphalus (penile length 5 cm), the testicular volume of 4 ml, sparse pubic and axillary hair growth (Tanner stage P II-III), but a normal sense of smell. He was tall above the age-related average, overweight, and bone age was moderately delayed. The testicles were descended at birth. Since 12 years of age, he noticed signs of adrenarche. He had no mirror movements, no renal or craniofacial abnormalities, and no signs of chronic disease. He had a low testosterone level, gonadotropins in the range of early puberty, and a normal inhibin B level. Magnetic resonance imaging at the age of 17 years demonstrated smaller anterior pituitary, but the rest of the pituitary function was normal. A clinical diagnosis of central hypogonadism was confirmed by genetic analysis. The patient achieved some partial gonadal development spontaneously and had a normal inhibin B level, while he received no pretreatment with gonadotropins. Given the unavailability of intermediate-acting intramuscular testosterone formulations, therapy with increasing doses of intramuscular long-acting testosterone undecanoate was initiated, which was much appreciated and desired by the patient and his parents. It is speculated that testosterone undecanoate could negatively impact final height achievement in younger boys [13]; however, given the patient's age and the achievement of most of his linear growth potential, negative implications of our decision were not expected. An incremental testosterone regimen by 3-monthly injection was started with a dose of 200 mg and gradually increased every 6 months until reaching 1000 mg over two years. During testosterone treatment, satisfactory virilisation with pubic hair and phallic development was achieved. However, there was no change in testicular volume. At the age of 19 years (after 2.5 years of treatment and after receiving three times the full dose of 1000 mg every 3 months), testosterone supplementation was interrupted by the patient, though he reported no adverse events related to the therapy. Six months later spontaneous increase in testosterone level to 12.7 nmol/L and an increase in testicular volume to 5 ml was noted. During the next 1.5 years, he had spontaneous pubertal development to achieve the testicular volume of 20 mL, with normal adult levels of gonadotropins and testosterone. His adult height (188.8 cm; Z-score 1.6) slightly exceeded his target height (185 cm; Z-score 1.1). The patient reported satisfaction with the treatment outcome. He was suggested to bank a sperm sample and warned about the possibility of relapse of hypogonadism. The mother's age of menarche was 14. Otherwise, the parents reported a normal course of puberty. A maternal aunt and one paternal aunt had infertility problems, but reported a normal course of puberty. Nevertheless, apart from the parents, genetic testing was not performed among other family members.

## DNA analysis

After obtaining written informed consent, targeted exome analysis was performed using the TruSight One sequencing panel (Illumina, San Diego, CA, USA) using MiSeq Desktop NGS Sequencer (Illumina, San Diego, CA, USA). Results were analysed with Illumina Variant Studio program and variants were confirmed by Sanger sequencing. Pathogenic *GNRHR* variants were tested in both parents by Sanger sequencing using in-house designed primers and a commercial kit BigDye Terminator v.3.1 Cycle Sequencing Kit and 3500 Genetic Analyzer capillary electrophoresis system (Life Technologies, Foster City, CA, USA).

Results of the molecular analysis of the proband revealed compound heterozygosity for two previously reported pathogenic missense variants in *GNRHR* gene, NM_000406.2:c.416G > A (NP_000397.1:p.Arg139His) and c.785G > A (p.Arg262Gln). According to the criteria of the American College of Medical Genetics (ACMG) the NM_000406.2:c.416G > A variant was classified as pathogenic and NM_000406.2:c.785G > A as likely pathogenic. The parents were heterozygous carriers (mother carrying p.Arg262Gln variant, father carrying p.Arg139His variant).

## Discussion and conclusions

Patients with CHH often report lasting psychological, emotional, social, and psychosexual difficulties [14]. Although infertility in CHH is generally well treatable using gonadotropin injections or GnRH pump therapy [2], a much lower percentage of men with CHH than expected father biological children [15]. One out of five patients experiences reversal of hypogonadism, with resulting gonadal maturation and the possibility of spontaneous fertility [12]. Spontaneous gonadal function could have positive implications on their self-esteem, partnerships, and family planning. The factors that pave a huge step between the treatable and healed are poorly understood. We present the clinical course of a patient with *GNRHR* deficiency who underwent complete gonadal maturation after being treated with testosterone.

Our patient was identified with two pathogenic *GNRHR* variants, both previously evaluated and reported in several CHH patients, each on a founder allele [16]. The p.Arg139His variant is a complete loss-of-function variant, eliminating GnRH binding activity [17]. The p.Arg262Gln variant leads to the reduced signal transmission, resulting in partial inactivation of the GnRH receptor [8].

Reversal can occur in patients with genetically confirmed CHH [12, 18–20]. CHH genes associated with cases of reversal are *ANOS1*, *CHD7*, *FGFR1*, *HS6ST1*, *NSMF*, *PROKR2*, *IGSF10*, *TAC3*, and *TACR3*. However, the *GNRHR* variants, including biallelic, have been described as leading variants among reversal cases, p.Arg262Gln being one of the more common variants associated with reversal [21–24]. A patient with the same combination of *GNRHR* variants, as identified in our patient, had a reversal of CHH after hormone replacement therapy was discontinued, similarly as in our patient, but after nine years of spontaneous function, he experienced a relapse of CHH [22]. The underlying mechanism of reversal is currently unknown. It usually follows sex steroid therapy, gonadotropin therapy, or pulsatile GnRH therapy [12], the common denominator being achievement of normal blood sex steroid levels [20]. Higher body mass index (BMI) negatively affected serum testosterone level after a reversal in monozygotic twin brothers [23].

Distinguishing CDGP from CHH reversal is challenging in patients with reversal before 20 years of age, as in our case. The biochemical tests have diagnostic specificity and sensitivity limitations to distinguish between CDGP and CHH. Our patient had a milder CHH phenotype with partial pubertal development before any therapy and inhibin B levels higher than those with severe CHH [2]. This is not unexpected; there is a good phenotype-genotype correlation in hypogonadism due to biallelic *GNRHR* variants [11]. Nevertheless, in a larger study, patients with partial puberty without micropenis or cryptorchidism did not reverse more commonly than patients with signs of severe CHH [12]. In view of this unpredictability and importance, supervised periodic treatment withdrawal every two years in patients with nCHH and Kallmann syndrome is suggested [20]. CDGP and CHH might lie on the different ends of the same spectrum of GnRH deficiency. However, studies investigating the genetic overlap between these two entities suggest a distinct genetic basis for CHH and CDGP [25]. Identifying CHH is clinically important since 13% of reversal cases in a larger study were followed by relapse [12], therefore sperm banking is a reasonable precaution procedure for patients with recovered gonadal function (20). Despite complete testicular development in our patient, follow-up for possible relapse of hypogonadism is warranted. Our report is limited by a short follow-up, as it will be interesting to observe if he can maintain hormonal balance over time in physiological values.

In conclusion, we emphasize the importance of awareness of the potential reversibility of CHH and knowledge of clinical signs of gonadal axis activation. Genetics helped distinguish CHH from CDGP in our case, as reversal occurred early, before 20 years of age.

## Data Availability

Data sharing is not applicable to this article as no datasets were generated or analyzed during the current study.

## Abbreviations

CHH: Congenital hypogonadotropic hypogonadism
GnRH: Gonadotropin-releasing hormone
GNRHR: Gonadotropin-releasing hormone receptor
CDGP: Constitutional delay of growth and puberty
nCHH: Normosmic CHH
LH: Luteinizing hormone
FSH: Follicle-stimulating hormone
GP: Greulich and Pyle
BMI: Body mass index
SDS: Standard deviation score
